# Time for Paradigm Shift in Anemia Assessment?

**DOI:** 10.1016/j.advnut.2024.100293

**Published:** 2024-10-03

**Authors:** Melissa F Young

**Affiliations:** Hubert Department of Global Health, Rollins School of Public Health, Emory University, Atlanta, GA, United States

**Keywords:** hemoglobin, anemia, assessment, global health

Anemia has been a persistent global health problem for decades impacting >269 million children and 539 million women, with a disproportionate burden among low- and middle-income (LMIC) countries [1]. Historically, most of the data used to generate global estimates of anemia in LMICs have been based on a single-drop capillary blood sample (finger/heal prick) and the use of point-of-care hemoglobinometers. However, this field-friendly and low-cost approach commonly used for hemoglobin assessment is currently being brought under question [[2], [3], [4]].

Karakochuk et al. [2] provide an insightful overview of key evidence and critical issues with hemoglobin assessment for population-level anemia surveys. The review summarizes the collective work of the Technical Expert Advisory group on nutrition Monitoring working group for anemia. The authors caution that use of single-drop capillary blood may introduce high levels of random error and thus lead to decreased accuracy and precision of population-level anemia estimates. Random error is particularly problematic as opposed to systematic error because it cannot be adjusted for and can be difficult to distinguish when both sources of error are present. In data simulations of the high rates of random error, a hemoglobin concentration of 115 g/L could be incorrectly reported as low as 95 g/L or as high as 135 g/L and population-level anemia prevalence could shift by 8 percentage points. In populations where the mean hemoglobin concentrations are near the cutoffs for anemia, the impact on anemia prevalence may be even larger. For example, in a case study among women in Uttar Pradesh, India (mean hemoglobin 123 g/L), the prevalence of anemia varied from 59% using capillary blood to 35% with venous blood [5]. Although we lack a global consensus on acceptable thresholds of error for hemoglobin assessment in surveys, this is clearly of public health significance and could drastically change the implications for anemia programs and policies within a country. However, it is important to note that some studies have reported lower variation in hemoglobin concentrations (within <0.5 g/dL) with different blood types and analytic approaches [6], and updated systematic reviews are under development. Others have questioned, “how good is good enough” [7], although the answer may be dependent on the purpose of the assessment and the level of precision required (national surveys, program evaluation, and referrals/clinical care). Best practices for hemoglobin assessment remains an active area of research, with several unanswered questions highlighted in the review, including whether we could optimize standard operating procedures (SOPs) for pooled capillary blood to minimize measurement error or apply corrections to adjust for the systematic error associated with the use of hemoglobinometers. In the meantime, government and program officials rely on accurate data for decision making and this review as well as growing evidence in the field suggests that it may be time for paradigm shift in anemia assessment [2,8,9]. This recommendation is in alignment with recently published WHO Hemoglobin Guidelines and Technical Brief [3,4].

A shift in anemia assessment practices (from capillary blood to venous blood) presents both critical challenges and new opportunities to advance anemia surveillance. First, there are significant implications for budget and field logistics. Venous blood collection requires a trained phlebotomist, and cold chain and automated analyzers are substantially more expensive than point-of-care hemoglobinometers [4]. There is a concern that the added cost and logistics may lead some counties to simply leave hemoglobin assessment out of national surveys and that could be a huge lost opportunity for tracking this important public health indicator. However, if cost or availability of automated analyzers is not feasible, venous blood in a point-of-care device is recommended. Second, some researchers have suggested that research on unintended consequences of switching to venous blood may be merited [6]. For example, there could be potential selection biases if there are differences in refusal rates by location, income, age (e.g., young children), or other factors. The implications of changes in anemia assessment are likely to impact lower resource areas the hardest and there is urgent need for investment in infrastructure, technical support, and capacity building to support this transition. Furthermore, there is a challenge in how we interpret historic data and anemia time trends with varying methodologies. Although many questions remain, one simple first step could be standardizing the reporting on hemoglobin methods going forward to include the following: *1*) blood source, *2*) analytic device, and *3*) reporting on SOPs and quality assessment as detailed in the recent Technical Expert Advisory group on nutrition Monitoring technical brief [4].

There are also clear advantages of revising hemoglobin assessment protocols. First, revisions and standardization of hemoglobin assessment will result in greater precision and accuracy of population-level anemia estimates. This is critical for correctly identifying the public health severity (mild, moderate, and severe public health problem) and for determining needed actions and level of investment. Poor-quality data and conflicting results can lead to confusion and mistrust of nutrition surveys. There is a need for high-quality data to inform decision making for programs and policies to reduce anemia. Second, this may be an opportunity to expand biomarkers beyond hemoglobin. Anemia etiology is complex and context specific and although hemoglobin assessment is essential for understanding the anemia burden, it may be insufficient alone to inform programs. Ideally, we can leverage the opportunity of obtaining a venous blood sample to include other key context-specific drivers of anemia (inflammation, micronutrient deficiencies, malaria, etc.) [10]. Poor measurement and lack of a multisectoral approach to anemia reduction programs in countries has resulted in insufficient global progress [11]. Going forward careful attention to compliance with SOPs and comprehensive assessment and reporting of hemoglobin data for quality is essential for data interpretation. Hopefully, the tide shifting to greater precision in anemia prevalence and etiology will help facilitate the needed change to meet global anemia reduction goals.

## Author contributions

The sole author was responsible for all aspects of this manuscript.

## Conflict of Interest statement

MFY was a member of the World Health Organization Guideline Development Group Member for Anemia. She receives funds from the Bill and Melinda Gates Foundation, Centers for Disease Control, National Institute of Health, and Emory University to support other ongoing research.

## Funding

The author reported no funding received for this study.
